# The hazard of using the Poisson model to cope with immortal time bias in the case of time-varying hazard

**DOI:** 10.1186/s12874-024-02396-y

**Published:** 2024-11-09

**Authors:** Federico Rea, Gabriella Morabito, Giovanni Corrao, Anna Cantarutti

**Affiliations:** 1 National Centre for Healthcare Research & Pharmacoepidemiology, at the University of Milano-Bicocca, Milan, Italy; 2 Department of Statistics and Quantitative Methods, Laboratory of Healthcare Research & Pharmacoepidemiology, Unit of Biostatistics, Epidemiology and Public Health, University of Milano-Bicocca, 8, Edificio U7, Milan, 20126 Italy

**Keywords:** Immortal time bias, Poisson model, Cox Model, Time-varying risk, Simulation study

## Abstract

**Background:**

A time-dependent analysis, usually by means of Poisson and Cox regression models, can be applied to prevent immortal time bias. However, the use of the Poisson model requires the assumption that the event rate is constant over time. This study aims to assess the potential consequences of using the Poisson model to cope with immortal time bias on estimating the exposure-outcome relationship in the case of time-varying risks.

**Methods:**

A simulation study was carried out. Survival times were assumed to follow a Weibull distribution, and the Weibull parameters were chosen to identify three different scenarios: the hazard of the event is constant, decreases, or increases over time. A dichotomous time-varying exposure in which patients can change at most once from unexposed to exposed was considered. The Poisson model was fitted to estimate the exposure-outcome association.

**Results:**

Small changes in the outcome risk over time (as denoted by the shape parameter of the Weibull distribution) strongly affected the exposure-outcome association estimate. The estimated effect of exposure was always lower and greater than the true exposure effect when the event risk decreases or increases over time, and this was the case irrespective of the true exposure effect. The bias magnitude was positively associated with the prevalence of and time to exposure.

**Conclusions:**

Biased estimates were obtained from the Poisson model to cope with immortal time. In settings with a time-varying outcome risk, the model should adjust for the trend in outcome risk. Otherwise, other models should be considered.

**Supplementary Information:**

The online version contains supplementary material available at 10.1186/s12874-024-02396-y.

## Introduction

Immortal time bias is a source of systematic uncertainty that can affect observational studies in which exposure can change during the follow-up [1]. It refers to a period during which the outcome cannot occur because of the exposure definition. For example, in pharmacoepidemiology studies, if a cohort of patients is followed from the hospital discharge and the exposure is a drug prescription, the time until the prescription is defined as immortal because exposed individuals have to survive until the treatment definition is fulfilled [2]. If this unexposed period is not correctly managed in the design or analysis, biased results will be obtained.

Albeit this bias was identified decades ago [3], it occurs frequently even now in many fields [4–7]. Several approaches have been proposed to prevent immortal time bias [8], including the adoption of a time-dependent analysis, the analysis of only subjects who have survived until a pre-defined time point (i.e. the so-called “landmark analysis”), and a time-matched analysis in which, for each exposed subject, an individual not (yet) exposed on that date is identified. To properly classify the immortal person-time by using a time-varying exposure, Poisson and Cox regression models can be used [9]. Although the Poisson regression model is frequently applied [10–14], its use requires the assumption that the event rate is constant over time [15], and, thus, the same during the exposed and unexposed periods. This principle, known as exchangeability, is an essential conditional for observing unbiased associations [16]. However, suppose the exposure follows a specific trend (e.g., all patients are unexposed at the start of the study and can switch to exposure during follow-up), and the risk of the outcome increases (or decreases) over time. In that case, the baseline risks are non-exchangeable, and thus confounding affects the measure of the exposure-outcome relation. To handle time-varying outcome risk when using the Poisson model, appropriate intervals of follow-up time in which the event rate is approximately constant should be identified.

With these premises, a simulation study was carried out to assess the potential consequences of using the Poisson model to cope with immortal time bias on estimating the exposure-outcome relationship without taking into account the time-varying outcome risk. By considering a dichotomous time-varying exposure in which patients can change at most once from unexposed to exposed, the exposure-outcome relationship estimate was investigated by (i) modifying the baseline risk over time, (ii) changing the true exposure effect, and (iii) varying the prevalence of and time to exposure. Finally, the association between antibiotics use during pregnancy and the risk of short-term neonatal outcomes was investigated using data from the Lombardy Region.

## Methods

### Survival and exposure times

This study was based on simulations. To simulate survival times in the setting of time-varying hazard, event times were assumed to follow a Weibull distribution. The Weibull is a common time-to-event distribution characterized by two parameters: λ (scale) and υ (shape). When υ < 1, the hazard of the event decreases over time; when υ > 1, the hazard of the event increases over time; when υ = 1, the hazard of the event is constant over time, and the Weibull distribution reduces to an exponential distribution. In our analyses, survival times were censored after five time units.

We consider only one type of time-varying exposure, i.e. a dichotomous time-varying exposure in which patients can change at most once from unexposed to exposed. To simulate the exposure status, exposure times were initially assumed to follow a Uniform distribution from 0 to 10. In further analyses, we modified the way to generate the exposure status (see below). If the exposure time was less than the survival time, the patient was considered exposed from the former time, and a new event time was simulated. The exposure effect was assumed to be constant over time. The true risk ratio of outcome in relation to exposure was denoted by RR_T_.

### Main scenarios

To assess the validity of estimates from the Poisson model, Weibull parameters were arbitrarily chosen as follows to identify three different scenarios:

- scenario A (the hazard of the event is constant over time): λ = 0.1 and υ = 1;
- scenario B (the hazard of the event decreases over time): λ = 0.75 and υ = 0.33;
- scenario C (the hazard of the event increases over time): λ = 1e-05 and υ = 7.

For each scenario, 1,000 samples of size 10,000 were drawn. A Poisson regression model was used to assess the exposure effect by estimating the risk ratio of outcome in relation to exposure (denoted by RR_P_). For each scenario, the median of estimated risk ratios of the 1,000 samples were calculated.

### Varying the parameter values

To investigate the ability of the Poisson model to obtain unbiased results in different settings, three analyses were performed.

First, to evaluate the validity of estimates according to the trend of the outcome hazard, simulations were carried out by varying the Weibull distribution parameters from scenario A to scenarios B and C as follows:

- from A to B: λ = 0.75 and υ ϵ (0.1,1),
- from A to C: λ = 0.1 and υ ϵ (1,3).

In this analysis, the exposure effect was set to RR_T_=0.75.

Second, to verify how the exposure effect affects the estimates of the Poisson model, the exposure effect (RR_T_) was made to vary from 0.5 to 2.

Finally, to assess the impact of exposure time and prevalence on the results, we changed the way to generate exposure status. To explore the influence of exposure prevalence, we set it to 15%, 25%, and 40%. With this aim, exposure status was simulated for each patient from a Binomial distribution with the abovementioned probabilities. In addition, to investigate the impact of exposure time, the mean time to exposure was made to vary from 0 to 5 time units. Exposure times were simulated from a Gamma distribution with scale parameter equal to 0.1 and shape parameter from 0 to 50. In this analysis, the exposure effect was set to RR_T_=0.75.

In all analyses, we assumed no confounding between exposure and outcome.

Statistical analyses were performed within the R software environment. The base code used for the present study is reported in the Supplementary Material 1.

### Real-world data

To investigate a real-world scenario in pregnancy, the healthcare utilization databases of the Lombardy Region (Italy) were analysed to identify all deliveries between 2007 and 2017. Antibiotics dispensed during the third trimester of pregnancy as well as neonatal outcomes (low birth weight and small for gestational age) were assessed. Women were defined as exposed to antibiotics use after the date of the first drug dispensing. More details on these data and on the criteria for selecting the study cohort are available in previous studies [17, 18]. The Poisson and the Cox models were fitted to estimate the exposure-outcomes associations.

## Results

### Main scenarios

**Fig. 1:**
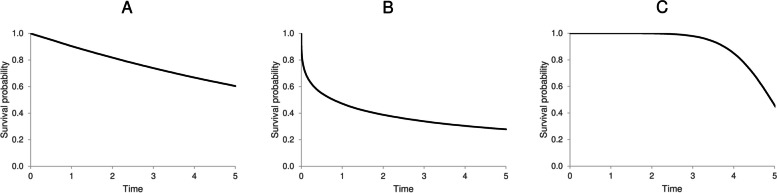
Kaplan–Meier curves of survival according to the three main scenarios Scenario A, in which the outcome hazard is constant over time, was obtained by assuming that survival times followed a Weibull distribution with λ = 0.1 and υ = 1. Scenario B, in which the outcome hazard decreases over time, was obtained by assuming that survival times followed a Weibull distribution with λ = 0.75 and υ = 0.33. Scenario C, in which the outcome hazard increases over time, was obtained by assuming that survival times followed a Weibull distribution with λ = 1e-05 and υ = 7

The survival curves estimated by means of the Kaplan-Meier method of the three scenarios are reported in Fig. 1. In scenario A, the outcome risk is constant; therefore, the proportion of patients who experienced the event is steady over time. Scenario B, in which the outcome risk decreases over time, is characterized by a higher proportion of patients who experienced the outcome at the beginning of follow-up. Finally, in scenario C, because the outcome risk increases over time, the number of patients who experienced the outcome quickly rises at the end of follow-up.

### Varying the baseline risk over time

**Fig. 2:**
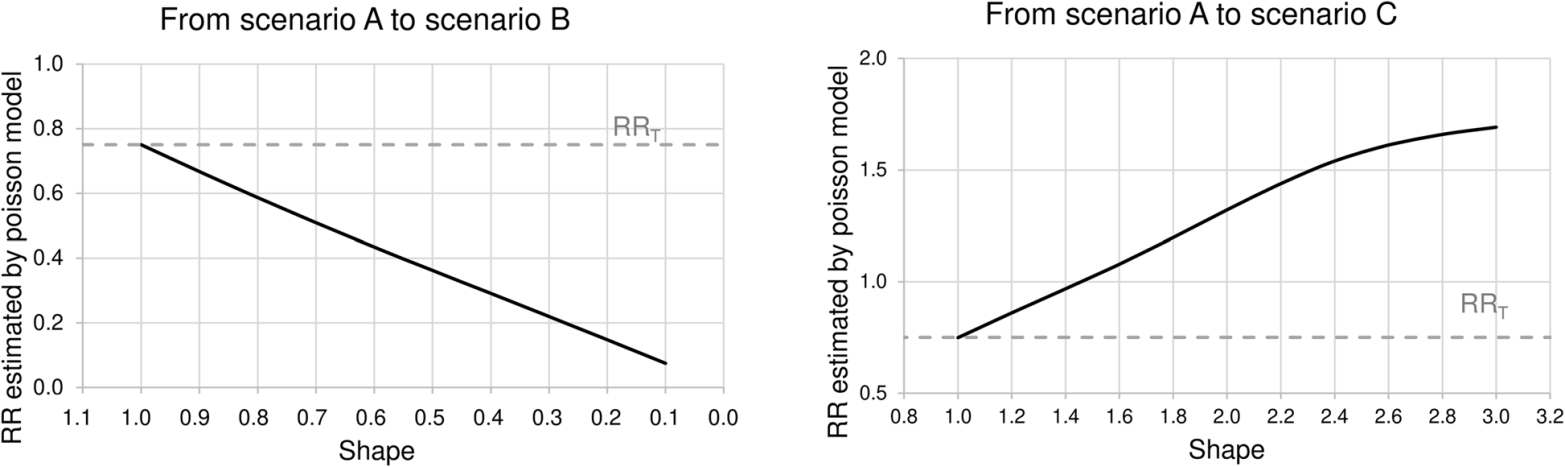
Risk ratios estimated by Poisson model (RR_P_) by varying the shape parameter of the Weibull distribution for generating survival times, that is by moving from scenario A to scenario B (left panel) and from scenario A to scenario C (right panel) The left panel was obtained by assuming that survival times followed a Weibull distribution with λ = 0.75 and υ ϵ (0.1,1). The right panel was obtained by assuming that survival times followed a Weibull distribution with λ = 0.1 and υ ϵ (1,3). The true exposure effect (RR_T_) was set to 0.75

Figure 2 shows the risk ratio estimated by the Poisson model (RR_P_) by varying the shape parameter of the Weibull distribution (i.e. by moving from scenario A to one of the other scenarios). Small changes in the shape parameter strongly affected the exposure-outcome association estimate, both towards scenarios B and C. For example, RR_P_ drops to 0.51 when υ = 0.7 and 0.22 when υ = 0.3 (Fig. 2, left panel). Conversely, RR_P_ increases to 1.08 when υ = 1.6 and 1.61 when υ = 2.6 (Fig. 2, right panel).

### Varying the exposure effect

**Fig. 3:**
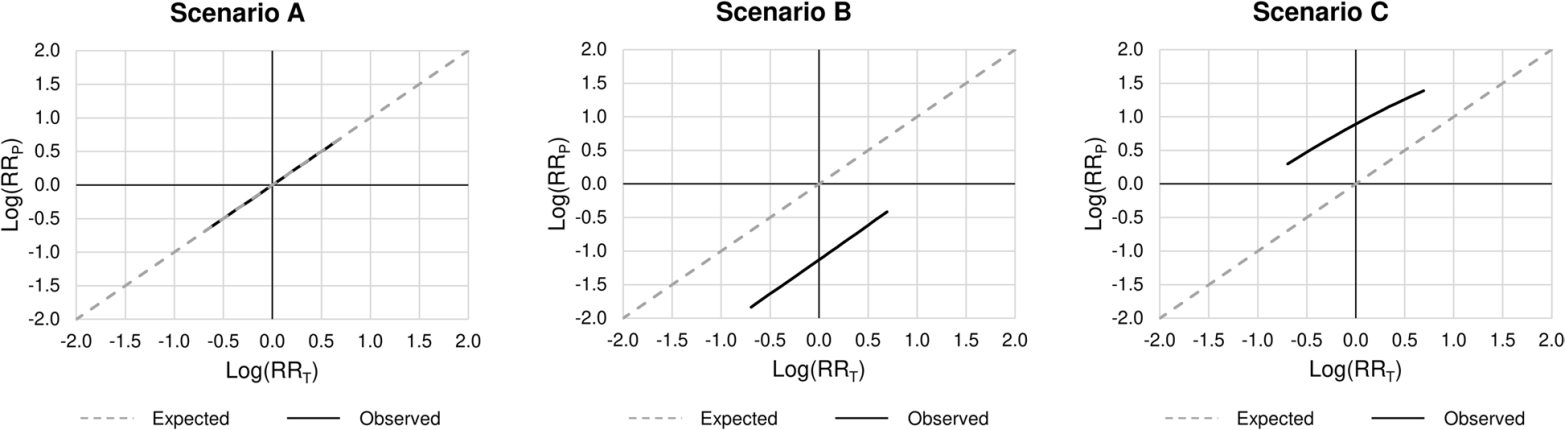
Risk ratios estimated by Poisson model (RR_P_) by varying the true exposure effect (RR_T_) according to the three main scenarios

The influence of the RR_T_ on the RR_P_ is reported in Fig. 3. In scenario A, the exposure-outcome association estimate from the Poisson model is always equal to the true exposure effect (Fig. 3, left panel). Conversely, the RR_P_ is always lower and greater than the RR_T_ in scenarios B and C, respectively, and the extent of the difference between the estimate and true effect is quite constant for each value of RR_T_ on the logarithmic scale (Fig. 3, central and right panel).

### Varying the prevalence of and time to exposure

**Fig. 4:**
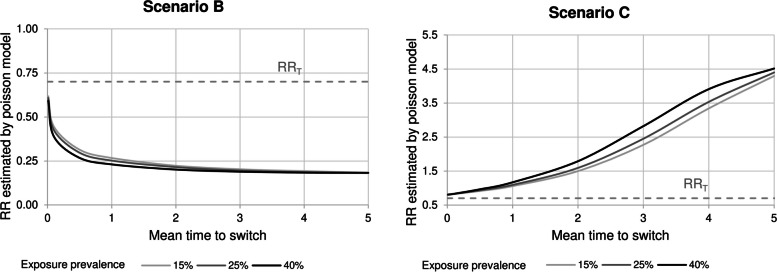
Risk ratios estimated by Poisson model (RR_P_) by varying the time to and prevalence of exposure Exposure status was simulated for each patient from a Binomial distribution with the following probabilities: 0.15, 0.25, and 0.40. Exposure times were then simulated from a Gamma distribution with scale parameter equal to 0.1 and shape parameter from 0 to 50. The true exposure effect (RR_T_) was set to 0.75

Figure 4 shows the impact of the exposure time and prevalence. The difference between RR_T_ and RR_P_ is greatest when the mean exposure time and prevalence are high. For example, in scenario B, RR_P_ is 0.27 (exposure prevalence = 15%) and 0.23 (40%) when the mean of time to exposure is 1 (Fig. 4, left panel), whereas, in scenario C, RR_P_ is 2.27 (exposure prevalence = 15%) and 2.82 (40%) when the mean of time to exposure is 3 (Fig. 4, right panel).

### Real-world data

The survival curves of the neonatal outcomes are reported in Supplementary Figure S1 (Supplementary Material 2). These outcomes occurred at the end of follow-up, as observed in the scenario C. Supplementary Table S1 (Supplementary Material 2) shows the association estimates between antibiotics use and neonatal outcomes according to the Poisson and Cox models. According to the Poisson model, antibiotics use was associated with a significantly greater risk of neonatal outcomes (low birth weight: +57%; small for gestational age: +73%). There was no association between antibiotics use and small for gestational age according to the Cox model, while a slightly increased risk was observed for low birth weight (+ 11%).

## Discussion

This simulation study shows the hazard of fitting the Poisson model to deal with immortal time bias in settings where the outcome risk varies over time and without taking into account this trend. Five main findings were provided. First, the estimate of the exposure-outcome association is already biased after minor changes in the trend of the outcome risk (as denoted by the shape parameter of the Weibull distribution). Second, the bias introduced using the Poisson model is not marginal since the exposure effect may be amplified, reduced, masked, or even reversed. Third, the direction of the bias is predictable. Indeed, the RR_P_ is always lower and greater than the RR_T_ when the event risk decreases or increases over time, respectively. Fourth, this was the case irrespective of the true exposure effect. Finally, the bias magnitude is positively associated with the prevalence of and time to exposure.

According to a meta-analysis of randomised controlled trials [19], antibiotics use is not associated with the risk of low birth weight and small for gestational age. Our real-world study in pregnancy shows that the estimates from the Poisson model suggested a non-marginal increased risk of neonatal outcomes among drug users (> 50%). Conversely, the Cox model showed no association between drug use and small for gestational age, whereas a significantly slight increased risk for low birth weight was observed, probably due to residual confounding [17]. Because the trend in the outcomes risk followed that of scenario C in our simulations, the overestimation of the exposure-outcome estimate by the Poisson model was expected.

In several settings, the outcome risk might vary over time. For example, the risk of several adverse events (including death and hospital admission) is higher just after a cardiovascular event [20], a fracture [21], and a transplant [22]. Otherwise, the risk of some maternal outcomes is higher at the end of the follow-up in pregnancy studies [23]. In addition, studies investigating lifetime survival are also characterized by an increased outcome risk over time [24]. In all these circumstances, the Poisson model wrongly estimates the true causal relationship between exposure and outcome. To obtain unbiased estimated, the model should adjust for the trend in outcome risk by identifying intervals of follow-up time in which the event rate is constant; however, the choice of the number of intervals as well as the position of the endpoints of each interval could be challenging [25]. Alternatively, other models, including the Cox model in which the outcome risk is left unspecified [18], should be considered.

Some limitations of the present study should be declared. First, the simulations were carried out by selecting one distribution for the survival times (i.e. the Weibull distribution) and changing the parameters in a small range of values (arbitrarily chosen to obtain three scenarios and to show the impact on the exposure-outcome estimate). Therefore, our simulations did not account for all possible scenarios. However, the study aimed to point out the risk of using the Poisson model in settings of time-varying hazards rather than to quantify the bias in several situations. Second, data were simulated assuming no confounding between exposure and outcome, and the impact of adjusting for covariates was not explored. Third, we also assumed that the exposure effect was fixed over time. Fourth, we investigated only one type of dichotomous time-varying exposure (allowing only one switch from unexposed to exposed), which does not cover different exposure patterns occurring in clinical practice. Finally, more complex real-world scenarios, such as competing risks, were not explored in our study. All these topics should be investigated in future studies.

In conclusion, the Poisson model provides biased estimates when outcome risk varies over time. In settings with a dichotomous time-varying exposure, the exposure-outcome association estimate is lower and greater than the true risk ratio when the outcome risk decreases or increases over time, respectively. Therefore, a careful assessment of intervals of follow-up time in which the outcome risk is constant is essential to fit the Poisson model.

## Supplementary Information

Supplementary Material 1.

Supplementary Material 2.

## Data Availability

No datasets were generated or analysed during the current study.
