# Supplementary material for: The hazard of using the Poisson model to cope with immortal time bias in the case of time-varying hazard
Source: BMC Med Res Methodol. 2024 Nov 9;24:272. doi: 10.1186/s12874-024-02396-y (PMC11549743; doi:10.1186/s12874-024-02396-y)
Supplement: Supplementary file 1 — Supplementary Material 1. [file 12874_2024_2396_MOESM1_ESM.pdf]

```
library(survival)

####################################################################################

# Define the input data.
# Change these data as described in the Methods section to obtain different scenarios

n <- 10000   # number of subjects
r <- 1000    # replications
scale <- 0.1  # scale parameter for time to event
shape <- 1   # shape parameter for time to event
HR <- 0.75   # hazard ratio for exposure
t.max <- 5   # time max to observation


####################################################################################

res <- data.frame(r=1:r,pois=rep(NA,r),cox=rep(NA,r))

for(q in 1:r){
 # create the data frame and generate the event and exposure times
 db <- data.frame(id = 1:n,
        exp=rep(0,n),
        tchange = runif(n, 0, 10),
        tfail = (-log(runif(n))/scale)^(1/shape)
 )

 # time to event and exposure
 db$tevent <- pmin(db$tfail, db$tchange, rep(t.max,n))

 # status
 db$fevent <- ifelse(db$tfail==db$tevent,1,0)
```

```
# censoring at the time of change
db1 <- db
db1$start <- 0
db1$stop <- db$tevent

# patients who switch
db2 <- db[which(db$tchange < db$tfail & db$tchange < rep(t.max,n)),]
db2$exp <- 1
db2$start <- db2$tchange

# event time after switch
u <- runif(1e+05)
tfail_exp <- (-log(u)/(scale*HR))^(1/shape)

for (i in 1:nrow(db2)){
  db2$tfail_new[i] <- sample(tfail_exp[which(tfail_exp>db2$start[i])],1)
}

db2$stop <- pmin(db2$tfail_new, rep(t.max,nrow(db2)))
db2$fevent <- ifelse(db2$stop < rep(t.max,nrow(db2)),1,0)

db3 <- rbind(db1,db2[,-c(8)])

# Poisson model
db3$logpy <- log(db3$stop-db3$start)
poisson.model <- summary(glm(fevent ~ exp + offset(logpy), family = poisson(link = "log"), data =
db3))
res[q,2] <- exp(poisson.model$coefficients[2,1])

# Cox model
cox.model <- coxph(Surv(start, stop, fevent) ~ exp, data=db3, timefix=FALSE)
```

```
  res[q,3] <- exp(cox.model$coefficients)

}

quantile(res$pois,probs = c(0.25,0.5,0.75))
quantile(res$cox,probs = c(0.25,0.5,0.75))
```
