# Supplementary material for: The hazard of using the Poisson model to cope with immortal time bias in the case of time-varying hazard
Source: BMC Med Res Methodol. 2024 Nov 9;24:272. doi: 10.1186/s12874-024-02396-y (PMC11549743; doi:10.1186/s12874-024-02396-y)
Supplement: Supplementary file 2 — Supplementary Material 2. [file 12874_2024_2396_MOESM2_ESM.docx]

**Supplementary Figure S1**. Kaplan–Meier curves of neonatal outcomes


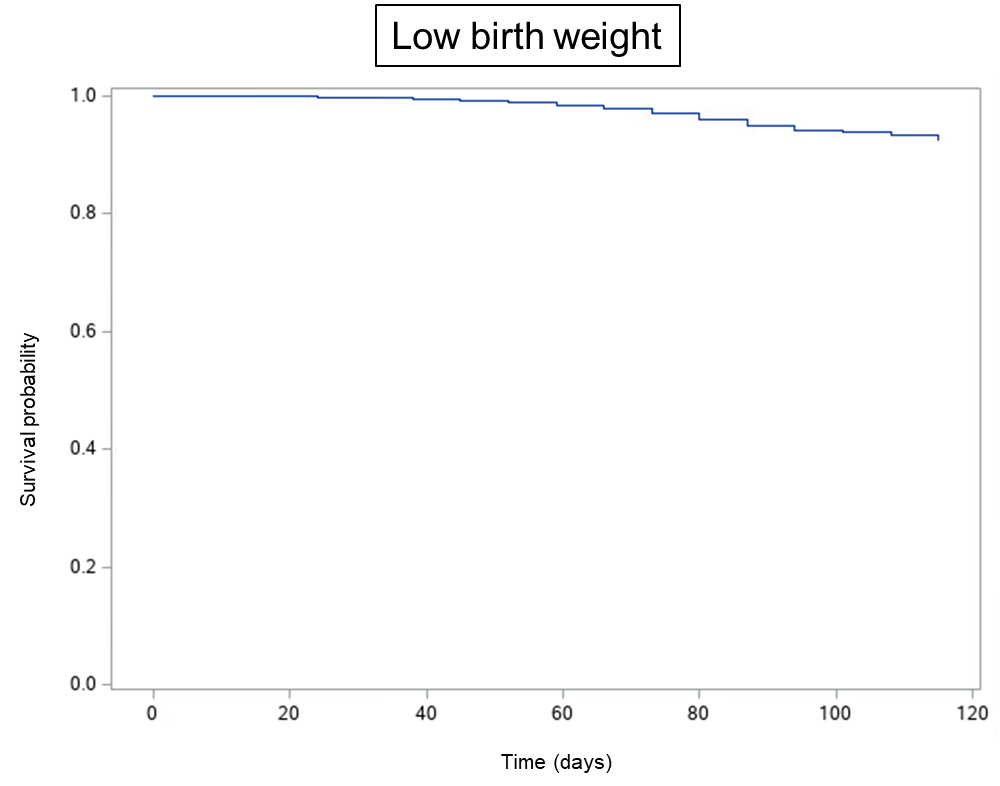


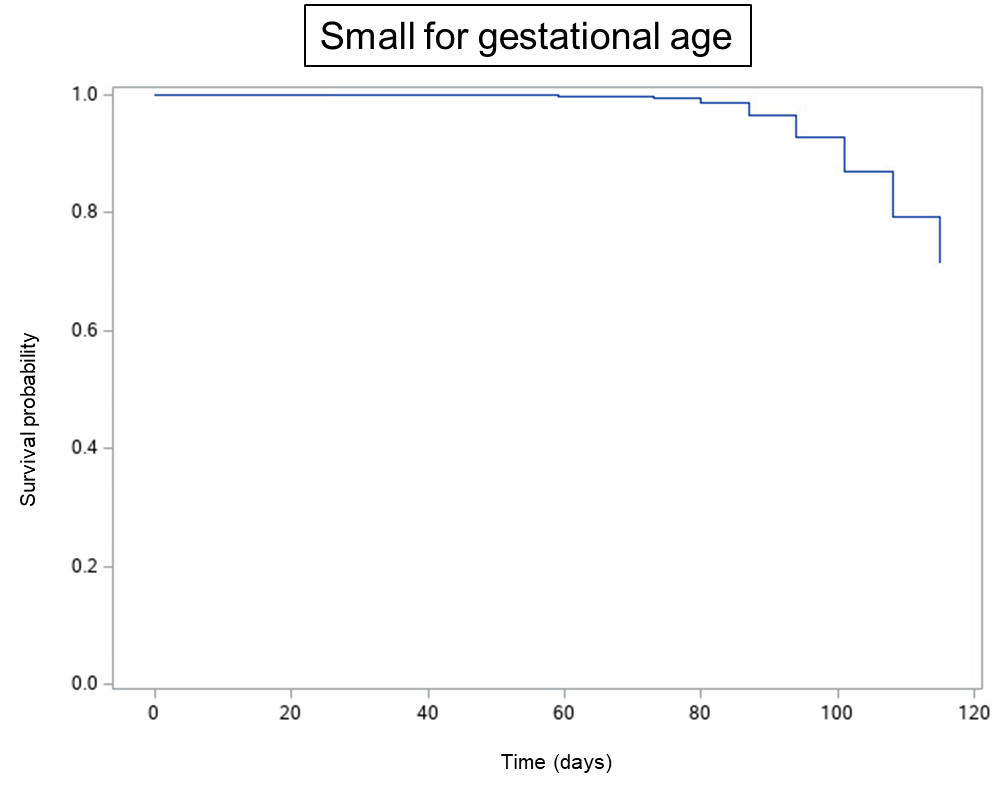


**Supplementary Table S1**. Association estimates, and 95% confidence intervals, between antibiotics use during the third trimester and neonatal outcomes

| **Outcome** | **Poisson model** | **Cox model** |
| --- | --- | --- |
| Low birth weight | 1.57 (1.50 to 1.66) | 1.11 (1.05 to 1.17) |
| Small for gestational age | 1.73 (1.67 to 1.79) | 0.97 (0.94 to 1.00) |

Adjusted models included sociodemographic features (i.e. age at delivery, nationality, marital status, education, employment, previous miscarriages, and parity), selected medical morbidities (i.e. diabetes, hypertension, preeclampsia, neuropathic, non-neuropathic, and other pain, obesity or overweight), and concomitant medications (i.e. non-steroidal anti-inflammatory drugs and triptans).
